# Urologic malignancy in renal transplant recipients: Analysis of OPTN/UNOS data from 2000 to 2022

**DOI:** 10.1186/s12885-026-16439-8

**Published:** 2026-06-27

**Authors:** Xiaowei Hao, Chenxi Ye, Wenhui Lai, Junnan Xu, Yangyang Wu, Yi Sun, Haigang Pang, Chao Lv, Kaikai Lv, Guorong Yang, Jie Wang, Yang Gao, Yin Lu, Shuai Huang, Zhenjun Luo, Jun Dong, Mingxing Zhao, Xu Zhang, Huaizhou Chen, Qing Yuan

**Affiliations:** 1 Department of Urology, Chinese PLA General Hospital, Beijing, China; 2 Medical School of Chinese PLA, Beijing, China; 3 Department of Urology, No.971 Hospital of PLA Navy, Tsingtao, Shandong China; 4 Hebei North University, Zhangjiakou, Hebei China; 5 The Organ Transplantation Department, Guilin No. 924 Hospital, Guilin, Guangxi China; 6 Department of Organ Transplantation, Shanghai Changzheng Hospital, Shanghai, China; 7 Department of Health Care, The Second Medical Center, Chinese PLA General Hospital, Beijing, China

**Keywords:** Urologic malignancy, Renal transplantation, End-stage renal disease, UNOS/OPTN, Risk factor

## Abstract

**Purpose:**

The increasing incidence of urologic malignancy after renal transplantation (RT) has become a leading cause of recipient mortality. However, no recent analyses have been performed to identify the risk factors for post-transplant urologic malignancy (PTUM) and to evaluate the effect of PTUM on RT outcomes.

**Materials and methods:**

This retrospective, population-based cohort study was based on Organ Procurement and Transplantation Network data.

**Results:**

A total of 268,606 recipients underwent RT from January 2000 to December 2019 and met the inclusion criteria. Of these, 2,079 (0.77%), 1,983 (1.20% of male recipients), and 846 (0.32%) patients were diagnosed with renal cancer (RCa), prostate cancer (PCa), and bladder cancer (BCa), respectively, after RT. Urologic malignancy was a major cause of patient death after RT (RCa: 41.5%, PCa: 23.5%, BCa: 50.4%). The 5-year survival rates of the four groups ranking from best to worst were as follows: [95% confidence interval, lower value–upper value], PCa, 93.3% [92.2%–94.4%]; cancer-free, 87.2% [87.0%–87.3%]; RCa, 87.2% [85.8%–88.7%]; BCa, 81.0% [78.4%–83.7%] (*P* < 0.001 for all, except cancer-free vs. RCa, *P* = 1.00).

**Conclusions:**

The effects of PTUM on RT outcomes differ depending on the type of malignancy. Thus, a personalized approach to screening may be an appropriate strategy to address the multitude of complex issues that RT recipients encounter.

**Supplementary Information:**

The online version contains supplementary material available at 10.1186/s12885-026-16439-8.

## Introduction

Renal transplantation (RT) is the most effective treatment for end-stage renal disease (ESRD), with a 5-year survival rate exceeding 90% [1, 2]. However, malignancy after RT is a leading cause of RT recipient death. The incidence of malignancy is high, and it can be extremely aggressive, necessitating heightened surveillance [3]. In fact, 56% of all deaths in patients with a functioning renal graft are due to malignancy, and more than 50% of RT recipients with malignancy lose their grafts within 5 years of diagnosis. The survival time of RT recipients who are diagnosed with malignancy (median survival, 2.1 years) is also significantly shorter than that of control subjects (median survival, 8.3 years) according to previous studies [4, 5].

Urologic malignancy is one of the most common post-transplant solid tumors, after non-Hodgkin lymphoma and lung cancer [6]. It mainly includes renal cancer (RCa), prostate cancer (PCa), and bladder cancer (BCa). Renal cancer is the second most frequent malignancy observed in patients who have undergone RT, of which renal cell carcinoma (RCC) accounts for 12% of post-RT malignancies, followed by non-melanoma skin cancer (20.5%). In the United States, RCa, PCa, and BCa were approximately 7-, 3-, and 2-fold more common in patients who had undergone RT than in the general population [7–9]. In a previous study, compared with patients on the RT waiting list, RT recipients had similar rates of BCa, a 39% higher rate of RCa, and a 21% lower rate of PCa [8]. While previous studies have characterized general post-transplant malignancy risks, the unique clinicopathological features of urologic malignancies remain underexplored.

The above-mentioned studies focused on the incidence of post-transplant malignancy or risk factors. However, these analyses were constrained by their insufficient sample sizes, which did not allow for adjustment of all confounders and/or identification of the risk factors for cancer incidence and prognosis in RT recipients. Thus, recent clinical studies examining urologic malignancy in RT recipients are still lacking. We conducted this retrospective, population-based cohort study using national data from the Organ Procurement and Transplantation Network (OPTN). This study identified the risk factors for three major types of PTUM and evaluated the effects of PTUM on the RT recipient and graft outcomes. Our findings will improve the understanding of PTUM and the development of strategies for tumor prevention and treatment.

## Methods

### Data source, study design and participants

**Fig. 1:**
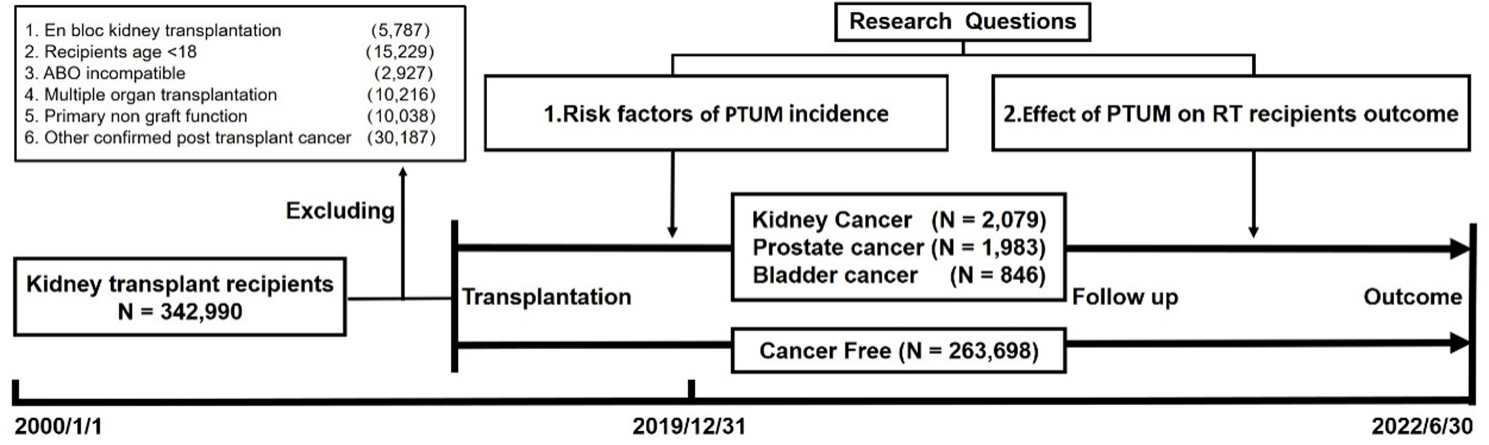
Flowchart of study cohort selection. PTUM, post-transplant urologic malignancy; RT, renal transplantation

We analyzed data from the OPTN Standard Transplant Analysis and Research file released in June 2022. This retrospective population-based cohort study included all adult kidney transplant recipients between 2000 and 2019. Recipients were followed through June 2022 unless they died, experienced graft failure, or were lost to follow-up, ensuring a minimum of 3 years of post-transplant observation. Recipients who were < 18 years old, ABO incompatible, received multiorgan transplants, primary non-functional grafts, other confirmed post-transplant malignancies, or those who received en bloc renal transplantation were excluded from the analysis (Fig. 1) This paper is the responsibility of the authors alone and does not necessarily reflect the views or policies of the Department of Health and Human Services, nor does the mention of trade names, commercial, or organizations imply endorsement by the U.S. Government.

### Exposure and outcomes classification and assessment

Time-to-outcome was defined as the date from RT until the date of the specified outcome (patient death or graft failure), censored for loss to follow-up, or the end of the study period. The outcomes included patient survival, graft survival, and death-censored graft survival. The patients were grouped into the RCa, PCa, BCa, and cancer-free (control) groups, based on the definition of “DeNovo Solid Tumor-Renal, Prostate and Bladder” in “MALIG_FOLLOWUP_DATA”. Post-transplant urologic malignancies were classified as recurrence in recipients with a history of treated urologic malignancy before transplantation, or as occurrence in recipients without any prior history of urologic malignancy. Renal cancer was defined according to the OPTN/UNOS variable “De novo solid tumor – Renal carcinoma.” Based on current WHO/ISUP classification, this category primarily represents renal cell carcinoma (RCC) arising from renal tubular epithelium and does not include upper tract urothelial carcinoma of the renal pelvis, which is classified separately as urothelial carcinoma. For incidence analyses, renal cancer (RCa) and bladder cancer (BCa) were calculated using the full cohort as the denominator, whereas prostate cancer (PCa) incidence was calculated in the male recipient subcohort only to avoid sex-related denominator bias. The ‘creatinine at most recent’ refers to the most recent creatinine measurement prior to PTUM diagnosis—extracted from the record immediately preceding cancer diagnosis for cancer groups, and from the last follow-up for the cancer-free group. This ensures that creatinine values represent pre-malignancy baseline renal function, appropriate for multivariate risk factor analysis.

### Statistical analysis

Patients’ demographic and clinical characteristics were compared using the chi-square test for categorical variables and the Student’s t-test for continuous variables; the distribution of the variables approximated normality. Patients were matched by the probability of urologic malignancy based on the multivariate logistic regression model with odds ratios (ORs). Missing data were handled using a two-step strategy. Variables with more than 5% missing values were excluded from multivariable analyses to ensure model stability. For variables with 5% or fewer missing values, missing entries were imputed using appropriate single-imputation methods. No multiple imputation was performed. The survival analysis is presented as Kaplan–Meier curves, and the data were compared using the log-rank test. All statistical analyses were performed using RStudio software, version 1.1.456 (RStudio, Inc., Boston, MA, US). A P value of < 0.05 was considered statistically significant, and all confidence intervals (CIs) were set at the 95% threshold. Descriptive statistics were used to summarize and present the data. Time periods are presented as median (interquartile range) and other continuous variables are expressed as mean ± SD.

## Results

From January 2000 to December 2019, 342,990 patients were registered for RT in the United Network for Organ Sharing (UNOS). A total of 268,606 RT recipients met the inclusion criteria, of which 2,079 (0.77%), 1,983 (0.74%), and 846 (0.32%) were diagnosed with RCa, PCa, and BCa, respectively, after RT (Fig. 1). Among them, 162,226 were male patients, for whom the respective numbers and proportions of RCa, PCa and BCa diagnoses were 1,470 (0.89%), 1,983 (1.20%) and 632 (0.39%).

Compared with patients in the cancer-free group, patients in the RCa, PCa, and BCa groups were older (49.6 vs. 53.1, 58.9, and 58.3 years, respectively), were more frequently male (59.9% vs. 70.7%, 100%, and 74.7%, respectively), were more frequently of White ethnicity (48.8% vs. 50.9%, 52.2%, and 76.6%, respectively), and more frequently had a transfusion history (14.1% vs. 15.9%, 15.9%, and 17.0%, respectively). They were also more frequently cytomegalovirus-positive (3.9% vs. 5.1%, 5.3%, and 5.1%, respectively); had a higher level of education (46.3% vs. 47.4%, 50.5%, and 49.3%, respectively); more frequently had the A blood group (36.9% vs. 37.4%, 38.9%, and 44.4%, respectively); and were more frequently treated with interleukin-2 receptor antibodies (25.7% vs. 27.7%, 31.2%, and 29.1%, respectively), cyclosporin (10.6% vs. 12%, 13.5%, and 11.6%, respectively), tacrolimus (61.8% vs. 71.3%, 69.4%, and 70.1%, respectively), or mycophenolic acid (MPA) (73.4% vs. 82.0%, 80.8%, and 80.3%, respectively). They also had lower panel reactive antibody sensitivity (≥ 30%; 20.8% vs. 17.4%, 14.2%, and 14.9%, respectively) and less frequently had T-cell depletion (58.8% vs. 54.4%, 51.5%, 51.7%, respectively) (Table 1).

**Table 1:** Characteristics of recipients and donors

Characteristics		Renal cancer (*n* = 2079)	Prostate cancer (*n* = 1983)	Bladder cancer (*n* = 846)	Cancer-free (*n* = 263698)	*p* value
Age at transplantation, yrs, mean (SD)		53.1 (11.8)	58.9 (8.4)	58.3 (12.0)	49.6 (13.8)	< 0.001
Sex, M, No. (%)		1470 (70.7)	1983 (100.0)	632 (74.7)	157,844 (59.9)	< 0.001
Ethnicity, No. (%)	White	1058 (50.9)	1035 (52.2)	648 (76.6)	128,709 (48.8)	< 0.001
	Black	686 (33.0)	691 (34.8)	109 (12.9)	71,157 (27.0)	
	Hispanic	224 (10.8)	191 (9.6)	48 (5.7)	43,423 (16.5)	
BMI > = 30 kg/m^2^, Y, No. (%)		738 (35.5)	584 (29.5)	208 (24.6)	82,196 (31.2)	< 0.001
Creatinine at most recent, mg/dL, mean (SD)		2.05 (1.44)	1.73 (0.77)	1.33 (0.42)	1.98 (1.38)	0.007
Cause of ESRD, No. (%)	Glomerular diseases	465 (22.4)	370 (18.7)	154 (18.2)	49,130 (18.6)	< 0.001
	Hypertension	589 (28.3)	547 (27.6)	167 (19.7)	58,587 (22.2)	
	Polycystic kidneys	132 (6.3)	243 (12.3)	80 (9.5)	22,690 (8.6)	
	Diabetes	415 (20.0)	438 (22.1)	193 (22.8)	66,541 (25.2)	
	Retransplant/graft failure	329 (16.1)	314 (16.2)	144 (17.2)	37,136 (14.1)	
Dialysis history, Y, No. (%)		1720 (82.7)	1534 (77.4)	652 (77.1)	216,940 (82.3)	< 0.001
Diabetes history, Y, No. (%)		555 (26.7)	610 (30.8)	253 (29.9)	84,417 (32.0)	< 0.001
Transfusion history, Y, No. (%)		331 (15.9)	315 (15.9)	144 (17.0)	37,087 (14.1)	< 0.001
HBV surface antigen, Positive, No. (%)		34 (1.6)	34 (1.7)	13 (1.5)	4465 (1.7)	0.982
CMV IgM, Positive, No. (%)		105 (5.1)	106 (5.3)	43 (5.1)	10,156 (3.9)	< 0.001
HIV antibody serum status, Positive, No. (%)		8 (0.4)	9 (0.5)	3 (0.4)	1726 (0.7)	0.197
Education level, College degree, No.(%)		986 (47.4)	1002 (50.5)	417 (49.3)	122,139 (46.3)	< 0.001
Private health insurance, No. (%)		741 (35.6)	830 (41.9)	355 (42.0)	95,435 (36.2)	< 0.001
Blood type, No. (%)	A	777 (37.4)	771 (38.9)	376 (44.4)	97,288 (36.9)	< 0.001
	B	244 (11.7)	263 (13.3)	110 (13.0)	34,151 (13.0)	
	AB	95 (4.6)	103 (5.2)	36 (4.3)	12,893 (4.9)	
	O	963 (46.3)	846 (42.7)	324 (38.3)	119,366 (45.3)	
Waiting time, yrs, median (IQR)		1.5 [0.5, 3.3]	1.3 [0.5, 2.9]	1.2 [0.4, 2.5]	1.3 [0.4, 3.0]	< 0.001
Dialysis duration, yrs, median (IQR)		2.1 [0.5, 4.6]	1.6 [0.0, 4.0]	1.3 [0.0, 3.1]	2.1 [0.4, 4.5]	< 0.001
KDPI, mean (SD)		0.40 (0.26)	0.42 (0.26)	0.40 (0.27)	0.40 (0.26)	0.022
Donor age, yrs, mean (SD)		40.2 (14.3)	42.1 (13.9)	40.6 (14.2)	39.5 (14.4)	< 0.001
Donor sex, M, No. (%)		1092 (52.5)	1024 (51.6)	440 (52.0)	139,178 (52.8)	0.736
Donor ethnicity, No. (%)	White	1502 (72.2)	1451 (73.2)	685 (81.0)	180,149 (68.3)	< 0.001
	Black	290 (13.9)	259 (13.1)	64 (7.6)	34,184 (13.0)	
	Hispanic	226 (10.9)	230 (11.6)	70 (8.3)	38,593 (14.6)	
Donor BMI > = 30 kg/m2, Y, No. (%)		549 (26.4)	499 (25.2)	212 (25.1)	68,613 (26.0)	0.725
Donor Type, Deceased, No. (%)		1352 (65.0)	1234 (62.2)	498 (58.9)	164,746 (63.6)	0.009
Deceased Donor Type, ECD, No. (%)		212 (15.7)	233 (18.9)	91 (18.3)	24,591 (14.7)	< 0.001
Deceased donor cause of death, No. (%)	Anoxia	322 (15.5)	267 (13.5)	122 (14.4)	47,149 (17.9)	< 0.001
	Cerebrovascular / Stroke	471 (22.7)	465 (23.4)	181 (21.4)	53,658 (20.3)	
	Head Trauma	512 (24.6)	461 (23.2)	173 (20.4)	61,617 (23.4)	
Donor smoking history, Y, No. (%)		323 (15.5)	299 (15.1)	138 (16.3)	35,798 (13.6)	< 0.001
Donor hypertension history, Y, No. (%)		919 (44.2)	864 (43.6)	323 (38.2)	141,261 (53.6)	< 0.001
Donor diabetes history, Y, No. (%)		719 (34.6)	672 (33.9)	255 (30.1)	122,629 (46.5)	< 0.001
Donor cancer history, Y, No. (%)		50 (2.4)	47 (2.4)	25 (3.0)	5995 (2.3)	0.574
HLA mismatch > = 3, Y, No. (%)		1653 (79.5)	1631 (82.2)	659 (77.9)	209,139 (79.3)	0.043
Panel reactive antibodies > = 30%, Y, No. (%)		361 (17.4)	281 (14.2)	126 (14.9)	54,758 (20.8)	< 0.001
Initial immunosuppression , No. (%)	IL-2 receptor antibody	576 (27.7)	619 (31.2)	246 (29.1)	67,642 (25.7)	< 0.001
	T-cell depleting	1132 (54.4)	1022 (51.5)	437 (51.7)	155,000 (58.8)	< 0.001
	Cyclosporin	250 (12.0)	267 (13.5)	98 (11.6)	27,843 (10.6)	< 0.001
	Tacrolimus	1482 (71.3)	1377 (69.4)	593 (70.1)	163,054 (61.8)	< 0.001
	MPA	1704 (82.0)	1602 (80.8)	679 (80.3)	193,563 (73.4)	< 0.001
	mTOR inhibitor	94 (4.5)	138 (7.0)	64 (7.6)	15,127 (5.7)	0.001

*yrs* years, *SD* Standard deviation, *M* Male, *Y* Yes, *BMI* Body mass index, *ESRD* End-stage renal disease, *ECD* Expanded criteria donors, *HBV* Hepatitis B virus, *CMV* Cytomegalovirus, *HIV* Human immunodeficiency virus, *IL-2* Interleukin-2, *KDPI* Kidney donor profile index, *HLA* Human leukocyte antigen, *MPA* Mycophenolic acid, *mTOR* mammalian target of rapamycin

In the donor cohort, the groups with malignancy consisted of more individuals of White ethnicity, more cases of cerebrovascular or stroke-related death, and more smokers, as well as fewer anoxia-related deaths and fewer patients with a history of hypertension or diabetes mellitus (Table 1).

Patients in the RCa group had the worst renal function (creatinine at most recent, 2.05 [1.44], mg/dL) and were most affected by glomerular diseases (22.4%) and hypertension-induced ESRD (28.3%), while they least frequently had private health insurance (35.6%), polycystic kidney disease (PKD) (6.3%), diabetes mellitus-induced ESRD (20%), and mammalian target of rapamycin (mTOR) inhibitor use (4.5%) (Table 1).

Patients in the PCa group were most frequently affected by PKD-induced ESRD (12.3%), had the poorest kidney donor quality (kidney donor profile index: 0.42), and had the greatest human leukocyte antigen mismatch (≥ 3) (82.2%) (Table 1).

The BCa group had the highest proportion of individuals of White ethnicity (76.6%), the lowest proportion of deceased donors (58.9%), the highest proportion of patients with mTOR inhibitor use (7.6%), and the fewest patients with a history of dialysis, as well as the shortest dialysis duration before RT. The proportion of expanded criteria donor (ECD) grafts was significantly higher in urologic cancer groups than in the cancer-free group (RCa, 15.7%; PCa,18.9%, BCa,18.3%; and cancer-free group, 14.7%; *P* < 0.001) (Table 1).

### Risk factors for PTUM incidence

**Fig. 2:**
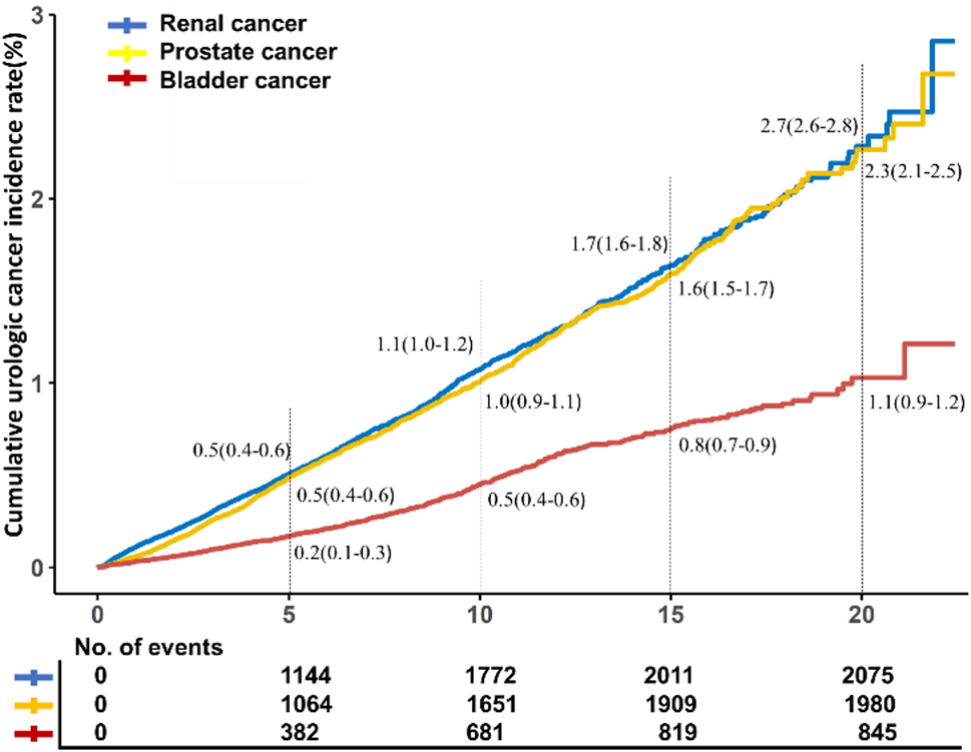
Kaplan–Meier curves of post-transplant urologic malignancy incidence by time after transplantation

Among the three types of urologic malignancy, the cumulative incidence ranking from highest to lowest was RCa, PCa, and BCa. The curve demonstrated a steadily increasing trend over time after RT, with minor fluctuations, which suggests that the incidence of new cases of PTUM in each group increased steadily with follow-up (Fig. 2).

**Fig. 3:**
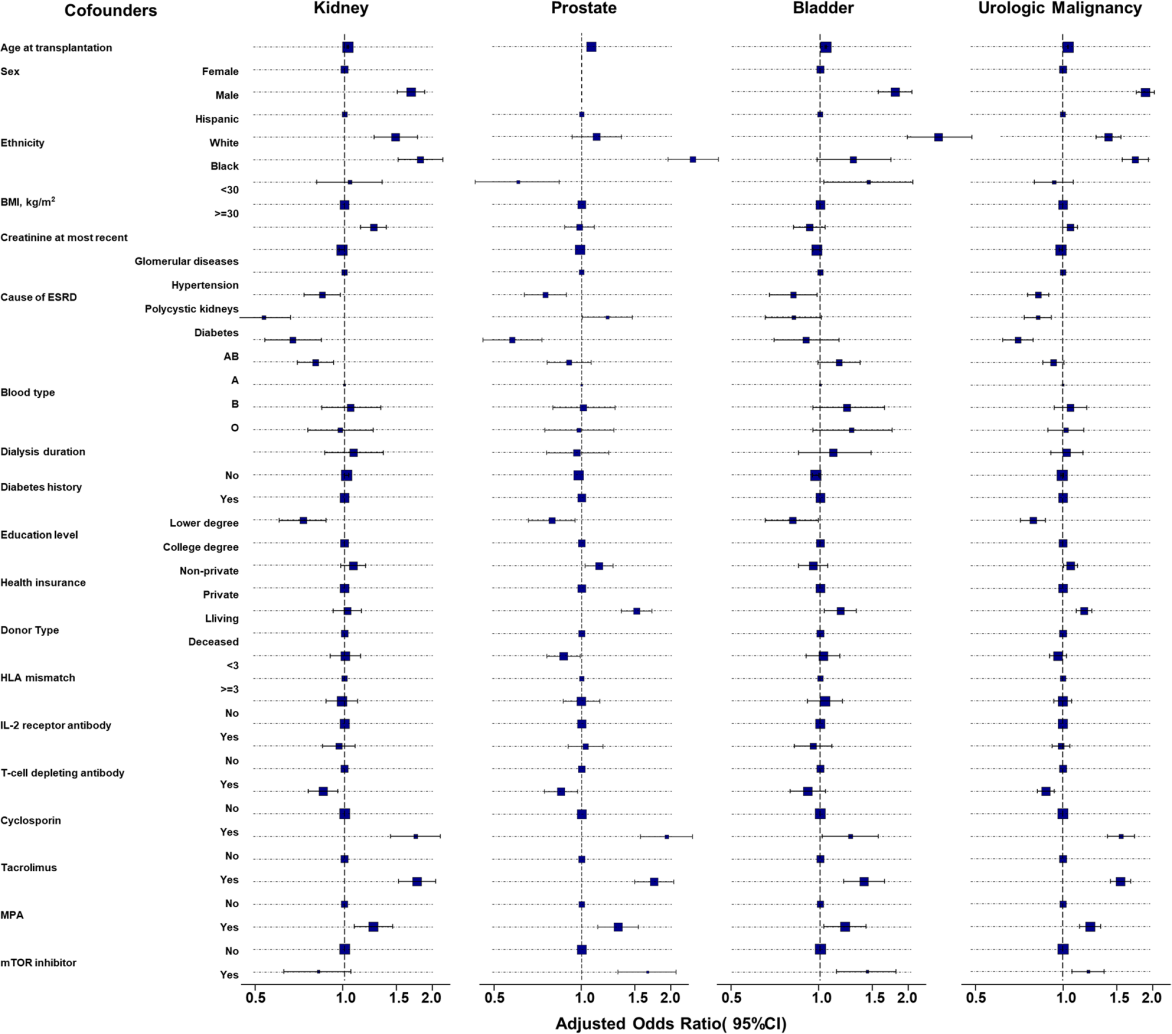
Multivariate analysis of risk factors for post-transplant urologic malignancy incidence

After allowing for the effects of the adjusted multivariate analysis, the logistic regression model suggested that recipient characteristics, including older age (adjusted odds ratio (aOR) > 1.0; *P* < 0.001), male sex (aOR [RCa, PCa, BCa, urologic malignancy] 1.66, not available, 2.15, 3.45; *P* < 0.001), cyclosporin use (aOR 1.72, 1.84, 1.36, 1.69; *P* < 0.05), tacrolimus use (aOR 1.74, 1.68, 1.56, 1.68; *P* < 0.001), and MPA use (aOR 1.25, 1.30, 1.29, 1.28; *P* < 0.001) were associated with an increased risk of urologic malignancy and each specific malignancy. Hypertension-induced ESRD (aOR 0.84, 0.77, 0.76, 0.80; *P* < 0.05) and diabetes history (aOR 0.73, 0.90, 0.75, 0.76; *P* < 0.05) decreased malignancy risk. Individuals of Black ethnicity were at a higher risk of urologic malignancy than individuals of White ethnicity, except for BCa (White vs. Black for each group: 1.47 vs. 1.78, 1.11 vs. 2.21, 3.34 vs. 1.40, 1.50 vs. 1.91) (Fig. 3, Table S1).

Diabetes mellitus-induced ESRD (aOR 0.67, *P* < 0.001; aOR 0.61, *P* < 0.001; aOR 0.86, *P* = 0.391; aOR 0.67, *P* < 0.001) and T-cell depleting antibody (aOR 0.85, *P* = 0.005; aOR 0.86, *P* = 0.014; aOR 0.88, *P* = 0.161; aOR 0.86, *P* < 0.001) were protective factors of urologic malignancy, RCa and PCa, except in the BCa group (Fig. 3, Table S1).

The use of mTOR inhibitors was a risk factor for PTUM, except in the RCa group (RCa: aOR 0.82, *P* = 0.129; PCa: aOR 1.60, *P* < 0.001; BCa: aOR 1.62, *P* = 0.002; urologic malignancy: aOR 1.26, *P* = 0.002). Body mass index (aOR 1.25, *P* < 0.001) and dialysis duration (aOR 1.02, *P* = 0.047) increased the incidence of RCa, while PKD-induced ESRD (aOR 0.54, *P* < 0.001) decreased the risk of RCa. RT recipients who had a higher level of education (aOR 1.13, *P* = 0.015) and had private health insurance (aOR 1.48, *P* < 0.001) were more likely to suffer from PCa. Conversely, recipients with a deceased donor (aOR 0.88, *P* = 0.033) or shorter dialysis duration (aOR 0.98, *P* = 0.025) had a lower incidence of PCa. Dialysis duration was a protective factor for BCa incidence (aOR 0.95, *P* = 0.007) (Fig. 3, Table S1).

### Effect of PTUM on RT outcomes

The temporal characteristics of PTUM development differed among the three malignancy types (Table 2). The median follow-up periods for PTUM patients were significantly longer than that of cancer-free recipients (8.9 [5.3, 12.8] years for RCa, 9.9 [6.6, 13.8] years for PCa, and 8.58 [5.1, 12.2] years for BCa vs. 5.8 [3.0, 9.5] years for cancer-free; *P* < 0.001). The median age at cancer diagnosis was 59.8 [50.6, 67.0] years for RCa, 65.1 [59.3, 69.9] years for PCa, and 66.8 [58.3, 72.6] years for BCa (*P* < 0.001). The median interval from transplantation to PTUM diagnosis was 4.5 [2.0, 8.1] years for RCa, 4.7 [2.5, 8.2] years for PCa, and 5.7 [2.7, 9.3] years for BCa (*P* < 0.001), indicating that BCa had the longest latency period after transplantation among the three malignancy types.

**Table 2:** Recipient status at follow-up with cause of death and graft failure

Characteristics		Renal cancer (*n* = 2079)	Prostate cancer (*n* = 1983)	Bladder cancer (*n* = 846)	Cancer-free (*n* = 263698)	*p* value
Follow-up period, yrs, median (IQR)		8.9 [5.3, 12.8]	9.9 [6.6, 13.8]	8.58 [5.1, 12.2]	5.8 [3.0, 9.5]	< 0.001
Age at diagnosed cancer, yrs, median (IQR)		59.8 [50.6, 67.0]	65.1 [59.3, 69.9]	66.8 [58.3, 72.6]	-	< 0.001
PTUM diagnosed interval, yrs, median (IQR)		4.5 [2.0, 8.1]	4.7 [2.5, 8.2]	5.7 [2.7, 9.3]	-	< 0.001
Post-transplant Recurrence of Pretransplant Malignancy, No. (%)		19 (2.2)	10 (0.5)	7 (0.3)	-	< 0.001
Delayed graft function, Y, No. (%)		360 (17.3)	311 (15.7)	115 (13.6)	45,929 (17.4)	0.005
Acute rejection, Y, No. (%)		177 (8.5)	47 (2.4)	19 (2.2)	25,622 (9.7)	< 0.001
Patient status, No. (%)	Alive	1138 (54.7)	1186 (59.8)	306 (36.2)	163,762 (62.1)	< 0.001
	Retransplant	90 (4.3)	48 (2.4)	8 (0.9)	15,100 (5.7)	
	Lost to follow up	83 (4.0)	118 (6.0)	24 (2.8)	31,730 (12.0)	
	Dead	768 (36.9)	631 (31.8)	508 (60.0)	53,106 (20.1)	
Cause of death, No. (%)	Other/Unknown	294 (38.3)	316 (50.1)	173 (34.1)	31,191 (58.7)	< 0.001
	Urologic cancer	319 (41.5)	148 (23.5)	256 (50.4)	807 (1.5)	
	Cardiocerebrovascular	70 (9.1)	81 (12.8)	37 (7.3)	10,961 (20.6)	
	Infection	80 (10.4)	82 (13.0)	41 (8.1)	9735 (18.3)	
	Graft failure	5 (0.7)	4 (0.6)	1 (0.2)	412 (0.8)	
Graft status, No. (%)	Functioning	968 (46.6)	1126 (56.8)	264 (31.2)	165,878 (62.9)	< 0.001
	Partial failure	688 (33.1)	570 (28.7)	463 (54.7)	48,058 (18.2)	
	Failure	423 (20.3)	287 (14.5)	119 (14.1)	49,762 (18.9)	
Cause of graft failure, No. (%)	Other/Unknown	1880 (90.4)	1839 (92.7)	790 (93.4)	233,435 (88.5)	< 0.001
	Rejection	177 (8.5)	125 (6.3)	51 (6.0)	27,177 (10.3)	
	Infection	2 (0.1)	2 (0.1)	0 (0.0)	247 (0.1)	
5-year survival rates, % (95% CI)		87.2 (85.8–88.7)	93.3 (92.2–94.4)	81.0 (78.4–83.7)	87.2 (87.0–87.3)	< 0.001
5-year cancer-specific mortality rate, No. (%)		315 (15.1)	122 (6.2)	237 (28.0)	-	< 0.001

*Y* Yes, *yrs* years, *IQR* Interquartile range

PTUM diagnosed interval, interval of urologic malignancy diagnosis after transplantation

UNOS does not provide standardized criteria for graft status categories, including Failed, Partial, and Functioning; therefore, we present this variable according to the original classification without imposing additional definitions

#### Patient survival

At the most recently reported follow-up, compared with the cancer-free group, the RCa, PCa, and BCa groups exhibited a significantly lower incidence of delayed graft function and acute rejection, a longer follow-up period, and a higher death rate (Table 2). Patients with BCa had the slowest malignancy onset speed (5.7 [2.7, 9.3] years). Patients with PCa had the longest follow-up period (9.9 [6.6, 13.8] years) and the lowest death rate (31.8%). As expected, BCa had the worst outcome, the shortest follow-up period (8.58 [5.1, 12.2] years), and the highest death rate (60%) (Table 2). A small proportion of recipients had recurrence of a pre-transplant malignancy after transplantation. The rates of post-transplant recurrence of pre-transplant malignancy were 2.2% in the renal cancer group, 0.5% in the prostate cancer group, and 0.3% in the bladder cancer group, with a significant difference among groups (*P* < 0.001).

**Fig. 4:**
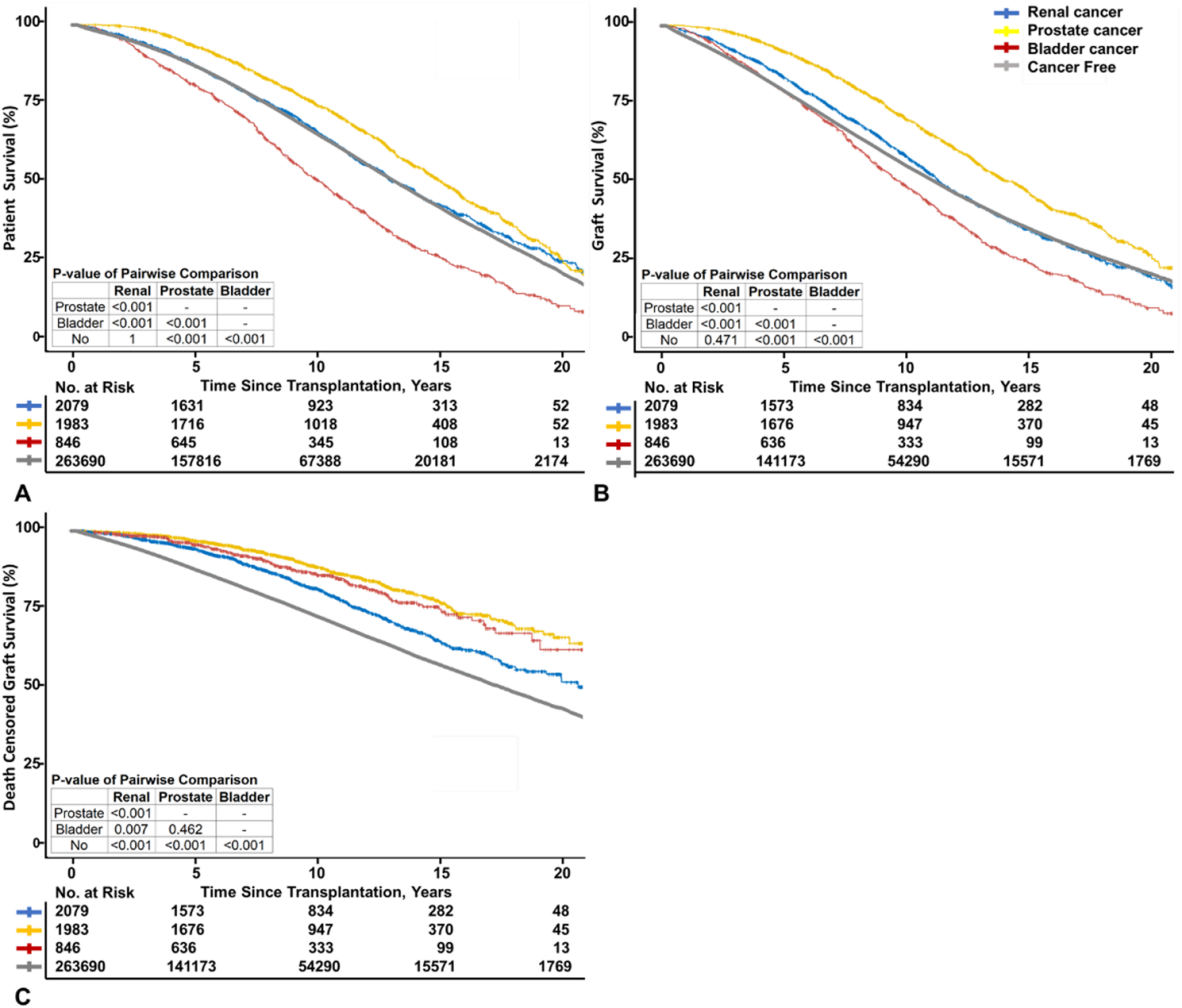
Kaplan–Meier survival curves of recipient and graft survival. Kaplan–Meier survival curves showing patient survival (**A**), graft survival (**B**), and death-censored graft survival (**C**) between PTUM and cancer-free renal transplantation recipients

At the most recently reported follow-up, the BCa group had a significantly higher rate of urologic malignancy mortality (50.4%) compared to the RCa group (41.5%) and PCa group (23.5%) (Table 2). The Kaplan–Meier curves show that PCa had the best outcome of the four groups in terms of patient survival, whereas BCa had the worst patient survival. The rate of patient survival was not significantly different between the RCa and cancer-free groups. The 5-year survival rates in the four groups ranked from best to worst were as follows: PCa, 93.3% [95% CI 92.2%–94.4%]; cancer-free, 87.2% [95% CI 87.0%–87.3%]; RCa, 87.2% [95% CI 85.8%–88.7%]; BCa, 81.0% [95% CI 78.4%–83.7%] (*P* < 0.001 for all, except cancer-free vs. RCa, *P* = 1.00) (Fig. 4A). In addition to overall survival, cause-specific outcomes were evaluated. The 5-year cancer-specific mortality rates were 15.1% in the RCa group, 6.2% in the PCa group, and 28.0% in the BCa group, with a significant difference among groups (*P* < 0.001) (Table 2). Consistent with these findings, urologic cancer was the leading cause of death in all three malignancy groups (RCa,41.5%, PCa,23.5%; and BCa,50.4%; respectively), whereas cardiovascular disease (20.6%) and infection (18.3%) accounted for a larger proportion of deaths in the cancer-free cohort (Table 2).

#### Graft survival

The RCa group had the highest graft failure rate (20.3%), while the cancer-free group exhibited the highest rate of graft function. The BCa group had the highest proportion of partial graft failures (54.7%). The major known cause of graft failure was rejection (Table 2).

As shown in the Kaplan–Meier curves, PCa (91.9% [95% CI 90.7%–93.1%]) showed the best graft survival, while BCa showed the worst graft survival (79.5% [95% CI 76.8%–82.3%]). The RCa (83.8% [95% CI 82.2%–85.4%]) and cancer-free (79.5% [95% CI 79.3%–79.7%]) groups displayed no significant difference (*P* = 0.471) (Fig. 4B). After death was censored from graft survival, PCa and BCa became the best two groups (*P* = 0.462), and the RCa group exceeded the cancer-free group (Fig. 4C).

## Discussion

In this study, we observed that the incidence of PTUM in the transplant cohort increased steadily over time after renal transplantation. These observations are consistent with previously reported elevated cancer risks in renal transplant recipients compared to general population estimates [10]. However, this study did not directly compare PTUM incidence with pre-transplant malignancy rates or with patients on dialysis, as such comparisons were beyond the scope of our analysis. The observed increase in PTUM incidence over post-transplant time may be attributed to multiple factors, including prolonged post-transplantation life expectancy, older age at transplantation, the effects of immunosuppressive therapy, and enhanced diagnostic surveillance during follow-up. In this large national cohort study of 268,606 renal transplant recipients, older age, male sex, Black ethnicity, and specific ESRD etiologies were associated with a significantly higher risk of PTUM. In terms of outcomes, patients with PCa demonstrated better patient and graft survival than patients with renal or BCa, patients with BCa had the worst patient survival, and patients with RCa and patients in the cancer-free group showed comparable patient survival. Importantly, our results highlight substantial heterogeneity across urologic malignancy subtypes after renal transplantation. RCa showed the highest cumulative incidence but limited impact on patient survival, PCa was associated with the most favorable patient and graft outcomes, whereas BCa carried the poorest patient survival and the highest cancer-related mortality. These differences suggest that a “one-size-fits-all” approach to post-transplant cancer surveillance may be inefficient, and that tumor-specific risk assessment is warranted.

Post-transplant malignancy is associated with several factors, including recipient age, sex, ethnicity, and use of immunosuppressive therapy. Conventional risk factors, such as older age and the male sex, are generally recognized as risk factors for urologic malignancy, and the sex proportion of PTUM coincides with that of the general population without RT [11–13]. We found that BCa burden of White ethnicity was higher than that of people of Black ethnicity, which is consistent with the higher bladder cancer burden of White ethnicity in the non-transplant population [14, 15]. Au et al. reviewed that overall cancer risk in White RT recipients is higher than that in other ethnicities [8, 16]. Nevertheless, risk (aOR) of RCa and PCa in RT patients with White ethnicity was lower than in Black ethnicity counterparts in the present study. It was also found that Black ethnicity has a significantly higher risk of RCa and PCa than other ethnicities both in renal transplant patients [17, 18] and in the non-transplant population [19, 20]. The disparity in incidence rates may be due in part to racial baseline risk, including genetics, exposures, diet, health conditions, and lifestyle [18, 21]. This finding suggests that for urological malignancy, the cancer risk attributable to transplantation is similar across racial/ethnic groups. Use of immunosuppressants is considered a risk factor for post-transplant malignancy due to their escape from immunosurveillance (immune escape) [22]. We found that initial maintenance therapy exhibited a significantly increased risk of post-transplant malignancy, while induction therapy decreased the risk of post-transplant malignancy. The use of mTOR inhibitors, which are immunosuppressants known for their anti-oncogenic effects [22], was not associated with a decreased risk of PTUM in the present study. This is supported by another OPTN study, which also found no strong evidence to suggest that sirolimus prevents post-transplant malignancy [23]. However, this may be because follow-up immunosuppressant regimen data in the database are not sufficiently detailed. Our finding that recipients who subsequently developed renal, prostate, or bladder cancer were more likely to receive kidneys from ECDs may partly reflect the biological vulnerability of these grafts. ECD kidneys are more prone to ischemia–reperfusion injury and carry a higher inflammatory burden, often requiring more intensive or complex immunosuppressive regimens, which together may foster a more tumor-promoting immune microenvironment in the allograft and the host [24]. Although ECD transplantation is associated with poorer graft function and an increased risk of certain malignancies [25], several studies have consistently shown that, for patients with long waiting times and substantial comorbidities, receiving an ECD kidney confers a clear survival advantage compared with remaining on dialysis [26]. Thus, in the context of organ shortage, ECD kidneys remain an acceptable option to expand the donor pool, provided that recipients are adequately counseled about the risks and are monitored carefully for post-transplant malignancies [27].

The cause of ESRD is another important factor related to PTUM development. The lower PTUM risk observed among recipients with PKD remains incompletely understood; however, PKD recipients in our cohort tended to be younger, less frequently Black, and had shorter dialysis exposure, which may partly explain this association. Future studies with richer phenotyping may clarify whether PKD-related biological mechanisms also contribute to reduced urologic cancer susceptibility.CKD itself is a recognized risk factor for RCC, and metabolic comorbidities common in CKD—including diabetes, hypertension, and obesity—have been linked to urologic malignancies [28]. Despite diabetes mellitus being linked to an increased risk of urologic malignancy in several studies, the improvement in metabolic abnormalities caused by anti-diabetes medications is associated with a decreased cancer risk [29, 30]. Interestingly, the dialysis duration, which is a risk factor for post-transplant malignancy according to a previous study [31], showed the opposite effect in patients with BCa in our study.

Consistent with previous studies, we observed a higher morbidity burden, but a moderate mortality rate, in RT recipients with renal cancer when compared with the non-transplant population (5-year cumulative incidence, 0.5% vs. 0.112%; 5-year survival rate, 87.2% vs. 75% [32]), suggesting that many cases may be detected at an earlier and more treatable stage. Therefore, post-transplant RCa does not obviously impact patient and graft survival. This might be due to the favorable prognosis of renal cancer and the option to return to dialysis to treat allograft cancer [33].

Notably, patient survival in the RCa group was comparable to that of cancer-free recipients, yet graft survival was modestly worse. In transplant oncology, post-transplant RCCs are broadly classified as recipient-derived de novo tumors or donor-related tumors (including donor-transmitted and donor-derived malignancies). Registry and pathological studies consistently indicate that the majority of de novo RCCs arise in the recipient’s native kidneys, whereas tumors originating from the allograft or transmitted from the donor are rare and account for only a very small fraction of all renal cancer events in OPTN/UNOS. Therefore, the inferior graft survival observed in our RCa group is unlikely to be predominantly driven by direct malignant involvement of the allograft, but rather by indirect mechanisms. Recipients who develop RCa tend to be older, with distinct ESRD etiologies and comorbidity profiles, that are themselves linked to chronic allograft dysfunction. In addition, a cancer diagnosis often prompts reduction or modification of maintenance immunosuppression, potentially increasing the risk of rejection and chronic allograft injury. Perioperative and cancer-related events (e.g., native nephrectomy, contrast-enhanced imaging, or related complications) may further destabilize allograft function and contribute to transient or sustained declines. Nevertheless, although uncommon, de novo RCC arising within the graft and donor-related RCC cannot be completely excluded in registry-based analyses and may have a disproportionate impact on graft outcomes in individual patients.

Post-transplant RCa is generally low-grade, and imaging surveillance after RT can identify kidney cancer at an early stage. A large, single-center study showed that a high proportion of patients with post-transplant RCa have RCC (91%), which has a favorable prognosis and a mean maximum tumor diameter of 3.3 cm (89% are smaller than 7 cm [pT1b]). Moreover, the cancer group exhibited favorable graft survival features compared with the cancer-free group due to better immunosuppression with lower risks of acute rejection and delayed graft function (DGF), as well as closer medical follow-up and thorough surveillance strategies [34].

In a previous study, an increase in the average age of RT recipients increased PCa cancer risk after RT [35]. In our study, patients with PCa had a favorable outcome compared with cancer-free patients after RT. This may be associated with their better health condition (less obese, favorable creatinine concentration) and more sufficient medical care (more private health insurance, PSA-based detection) in the PCa group. A systematic review showed that treatment for PCa after RT does not seem to have worse oncological outcomes or higher complication rates than non-transplant populations [36].

Although BCa was not as common as RCa or PCa in the present study, in RT recipients, it had a significantly more devastating patient prognosis. A potential extended life expectancy is determined by patient survival rather than graft failure. Compared with previous studies, patients in the present study were older at BCa diagnosis. A study performed in the US performed from 1964 to 2002 showed that the age at transplantation was 40 years, and the interval between transplantation and diagnosis was 5.3 years in patients with post-transplant BCa [37]. Another study performed in Ireland, which involved 3,688 RT recipients, indicated that the mean age at the time of bladder cancer diagnosis was 55.7 years, and the mean interval between transplantation and BCa diagnosis was 8.6 years [38]. However, compared with non-transplant patients, patients with BCa were younger at diagnosis (66.8 vs. 70–85 years) [39, 40] and had a better 5-year survival rate (81.0% [78.4%–83.7%] vs. 70% [60%–80%]) [41].

Several reports suggest that BCa in RT recipients is more likely to be diagnosed at an advanced stage, with higher proportions of muscle-invasive disease and metastatic presentation, potentially due to accelerated tumor growth under long-term immunosuppression and delayed recognition of hematuria or urinary symptoms. These aggressive clinicopathologic features—together with limited systemic options in transplant recipients (e.g., cisplatin-based chemotherapy constrained by nephrotoxicity and immune checkpoint inhibitors by concerns for allograft rejection)—likely contribute to the inferior survival and higher cancer-specific mortality observed in our cohort [42, 43]. Conventional systemic treatments for advanced BCa, such as cisplatin-based chemotherapy, have demonstrated survival benefits in the general population but are often limited in transplant recipients because of nephrotoxicity [44–46]. Although novel targeted and immune-based therapies have been approved for several indications in advanced BCa among non-transplant patients, their use in transplant recipients remains controversial due to potential interactions with immunosuppressive therapy and the risk of allograft rejection [47, 48].

The present study provides valuable insights into the screening, diagnosis, and treatment of PTUM, particularly in terms of risk stratification and individualized treatment [49]. Although cumulative incidence increased steadily over time, no acceleration in incidence rate was observed. Our findings suggest that RCa and PCa do not significantly impact patient and graft survival, as shown in our study. Therefore, maintaining a functioning allograft should be prioritized over PTUM, as the impact of PTUM on recipient and allograft survival is highly variable. Despite cancer after transplantation being a major cause of patient death and graft loss, the impact of PTUM on long-term survival differs considerably across tumor types [50]. Our results also highlight important differences in post-transplant malignancy outcomes across urologic cancers. While RCa patients showed survival rates comparable to cancer-free recipients, graft survival was modestly worse, likely due to indirect factors such as older age, comorbidities, and immunosuppressive therapy rather than direct malignancy involvement of the allograft. Despite the challenges with immunosuppressive therapy and potential delayed detection, PCa demonstrated better graft survival compared to RCa. These findings suggest that post-transplant urologic malignancies require tailored management strategies, including careful monitoring, potential adjustments in immunosuppressive therapy to manage cancer treatment while mitigating rejection risks, and individualized screening during early follow-up, particularly for high-risk patients, as routine screening for PTUM may not be necessary for all renal transplant recipients.

A preliminary risk-adapted screening framework could be considered to provide a more suitable and cost-effective approach to managing cancer treatment while mitigating rejection risks: (1) Identify higher-risk recipients using readily available variables (older age, male sex, Black ethnicity, longer dialysis exposure, higher BMI, specific ESRD etiologies, and intensified maintenance immunosuppression); (2) Apply tumor-specific surveillance—e.g., periodic renal imaging for recipients at higher risk of RCa, PSA-based screening within the male subcohort for PCa, and hematuria-triggered evaluation (urinalysis/urine cytology and cystoscopy where appropriate) for patients at elevated risk of BCa; and (3) Maintain long-term vigilance, as incident PTUM accumulated steadily over time rather than being confined to the early post-transplant period. This approach is intended as a pragmatic starting point for clinical translation and should be refined and validated in cohorts with more detailed tumor and treatment data.

The present study has several potential limitations that should be noted. First, immunosuppressant use was among the most important factors due to its influence on cancer. Nevertheless, the immunosuppressant regimen used during follow-up is insufficiently detailed in the UNOS registry. Second, due to the lack of data granularity, particularly as it relates to urologic malignancy details, such as grade, stage, time from surgery, and specific cancer treatments received, such as chemotherapy or radiation therapy, we were unable to include treatment-related variables. Although post-transplantation RCa was selected among other de-novo cancers, it remains difficult to distinguish renal cancer in a native kidney from RCa in an allograft. In addition, histological subtype is not specified in the OPTN/UNOS registry. Although “renal carcinoma” in contemporary oncologic classification refers to RCC and does not encompass upper tract urothelial carcinoma, the inability to distinguish RCC from rare renal pelvis urothelial tumors and to determine native-kidney versus allograft origin represents an inherent limitation of registry-based analyses. Third, donor-related cancer was not included as an exclusion criterion because the registration of this information only commenced in 2015. Fourth, the most common known cause of graft failure in all malignancy groups was rejection; however, the rate of unknown causes exceeds 90%. This prevents the evaluation of graft failure outcomes that may develop due to the PTUM. Finally, despite the relatively large sample size in this study, we lacked information on cancer risk after transplant failure and were limited in our ability to avoid loss to follow-up. These limitations may have led to underestimation of PTUM-related mortality in RT recipients.

In conclusion, the findings of this study provide a comprehensive description of PTUM risk factors and outcomes. RT recipients who are of an older age, male, of Black ethnicity, as well as those with certain causes of ESRD, and previous use of MPA, may be at a higher risk of PTUM. The effects of PTUM on RT outcomes differ depending on the type of malignancy; specifically, RCa and PCa have a limited impact, while bladder cancer is detrimental. Consequently, given the differences in the individual risk of PTUM and overall prognosis, a personalized approach to screening may be an appropriate strategy to address the multitude of complex issues that RT recipients encounter. Further study is necessary to clarify how to effectively intervene in cases that are at a high risk of PTUM.

## Supplementary Information

Supplementary Material 1. Supplementary Table 1. Multivariate analysis of risk factors for post-transplant urologic malignancy incidence.

## Data Availability

Publicly available datasets were analyzed in this study (https://optn.transplant.hrsa.gov/data/). The corresponding authors declare that the data supporting the findings of this study are available and will be provided upon request.
